# From Healing the Whole Person: An Argument for Therapeutic Touch as a Complement to Traditional Medical Practice

**DOI:** 10.1100/tsw.2006.348

**Published:** 2006-08-25

**Authors:** Marlene B. Huff, Kimberly K. McClanahan, Hatim A. Omar

**Affiliations:** Division of Adolescent Medicine, Department of Pediatrics, University of Kentucky, Lexington, KY, USA

**Keywords:** therapeutic touch, alternative treatment, nursing, U.S.

## Abstract

The growing popularity and use of therapeutic touch (TT) is an issue that has generated controversy and concern within the medical community. While anecdotal and traditional scientific evidence suggest that TT would be an advantageous addition for clinics and hospitals to include in their armamentarium of complementary interventions within the realm of traditional medicine, TT has not become widely available in the U.S. One reason for the lack of availability may be the dearth of conclusive scientific support for TT's efficacy and, therefore, its inclusion in clinic and hospital treatment planning would give it the appearance of legitimate practice, which it may not yet deserve. Whether or not deserved, if TT were added to hospital and clinic treatment protocols without substantial scientific support, it would be thought to have the implicit support of the scientific community, at which point the question of its efficacy would be moot in the minds of many people; thus patients would utilize it, because they *believe* it works rather than because it works. Since TT has not yet been scientifically proven as per Western standards, leaders of the health care community are likely wary of lending support to TT at this time. If TT can be found to be a scientifically sound therapeutic technique, then it will be more readily accepted in the health care community. This paper reviews TT.

## INTRODUCTION

Therapeutic touch (TT) is defined by the Nurse Healers-Professional Associates[1] as "an intentionally directed process of energy exchange during which the practitioner uses the hands as a focus to facilitate the healing process," (p. 23) although most TT does not involve actual physical contact, and the hands of the practitioner are held a few inches away from the body. Only when TT is used in conjunction with massage therapy is there physical contact between the health care professional and the patient. Krieger[2], whom many consider the founder of one of the most popular types of TT, further described the technique as a contemporary interpretation of ancient healing practices and complementary to traditional medicine. In recent years, though, TT has sometimes been viewed as competitive with, rather than complementary to, more traditional medical interventions. Thus, concerns have been raised about TT's efficacy as well as the economic impact it would have on traditional medical practice were TT to be utilized instead of traditional medical interventions. Given that TT has been seen as a competing treatment modality within the traditional medical community, its acceptance by some has contributed to the overall tension between what techniques are defined as “traditional” vs. “holistic” in nature. This sense of competition, combined with an ongoing lack of traditional experimental data “proving” that TT increases quality of life and promotes healing, has marginalized TT as a valid complement to traditional medical practices. Nonetheless, despite the absence of traditional scientific indicators that TT has healing qualities, it is practiced worldwide by all kinds of “alternative” healers. In fact, over 100,000 people have been trained in the TT technique, including at least 43,000 health care professionals. About half of those trained in TT actually practice it on a routine basis. As of 1988, TT was taught in 80 colleges and universities in the U.S. and in 75 countries outside of the U.S.[3]. TT is thought to be the most recognized technique used by practitioners of holistic nursing[4].

While the effectiveness of TT is only remotely understood using the traditional scientific approach, does that mean that TT has no place in traditional medicine? Based on available studies, we argue that TT has found an appropriate home in holistic medicine as a complement to traditional medical practices and can be useful in restoring quality of life to persons whose bodies are in the process of healing from trauma (either accidental trauma or the more typical bodily traumas brought on by everyday stress). Given an accurate understanding of TT techniques and the current state of research surrounding its effectiveness, the practice of TT belongs in the realm of traditional medicine, much like other nontraditional therapies that are holistic in their approach to maximizing overall health.

## DIMENSIONS OF TOUCH

What does touch mean to the practitioner who uses it to communicate care? Barnett[5] has identified five broad communication categories that have explored the dimensions of touch: (1) the mechanics of communication, (2) touch as a means of communicating, (3) touch as a basis of establishing communication, (4) touch as a means of communicating emotions, and (5) touch as a means of communicating ideas. Weiss[6] takes these dimensions one step further and attempts to identify the human responses associated with the type of communication elicited by touch, which includes developmental, interpersonal, and psychological responses. Weiss[7] also distinguishes between six tactile symbols in the act of TT: duration, location, action, intensity, frequency, and sensation. Finally, Karr[8] believes that TT can take the form of being spontaneous, pragmatic, or silent when providers use it to communicate care.

Weber[9] has proposed distinctions among three different touch models that lend a structure through which the various aspects of touch described herein can be assimilated: (1) physical-sensory model, (2) psychological-humanistic model, and (3) the field model. These models are often confused as all denoting TT, but this is not the case. Touch is defined as “to be in contact with” in the physical-sensory model, “to reach and to communicate” in the psychological-humanistic model, and “to lay hand or hands on” in the field model. The field model is the only one accepted as denoting valid TT. Randolph[10], Snyder[11], and Routasalo[12], among others, agree that there is a difference between physical and therapeutic touch, although little is empirically understood about these differences.

There seems to have developed some level of consensus regarding the components of touch. Almost all studies, for example, have accepted TT as being a nonverbal, nonvocal form of communication. In the majority of studies found in the current literature, touch has been conceptualized as physical contact[13,14,15,16,17], a physical response[18], tactile comfort[19], or tactile stimulation[20,21,22, 23,24]. The literature surrounding TT has also examined touch as behavior[25,26,27], while Estabrooks and Morse[28] described TT from the perspective of patients as they defined it. These studies have been both qualitative and quantitative in nature.

Despite the array of nebulous meanings attached to TT, many of the original practitioners indicated no particular need for a precise definition of the term. In fact, the founders of TT were primarily nurses and believed that the palms of the hand were chakras (a chakra is a point of energy associated with various parts of the body, emotions, desires, thoughts, powers, and health) that could be used to channel healing energy. The practice of TT is based on the Eastern medical paradigm, which greatly values the spiritual and emotional status of the person and does not perceive the necessity of any particular physical or chemical interventions (e.g., surgery, drugs)[29] or understanding of precise techniques. This is in sharp contrast to Western methods, which often require an understanding of every aspect of the medical process (including the development of new drugs or surgical procedures) through replications and generalizations to groups of individuals suffering from a specific malady.

## THE DIMENSIONS OF THERAPEUTIC TOUCH

TT is set apart from traditional medicine, as well as many other holistic healing modalities, by its emphasis on the healer's intention, not the procedure(s) that lead to the healing. In fact, the TT hypothesis and the entire practice of TT rest on the idea that the patient's energy is detected and intentionally manipulated by the practitioner.

There are a number of TT systems in use today, two of which are Krieger's method of TT[3,29,30] and Reiki's healing techniques[31,32]. Krieger's and Reiki's TT models are similar in the outcomes that are cited for most bio-field therapies: relaxation, decreased anxiety, feelings of well-being, and healing, and even the minimal distinctions between the models listed in Table 1 are currently subject to discussion among scholars and practitioners alike. The differences between Krieger's and Reiki's philosophies on TT appear to primarily lie in the philosophical underpinnings of the disciplines and the way practitioners are trained. For example, Krieger's philosophy of TT maintains that anyone can perform energy healing. Practitioners then learn the techniques and how to connect with the patient. The Reiki philosophy also agrees that anyone can perform energy healing. The Reiki attunement process, however, claims to "open" healers and "facilitate" the patient-practitioner connection. Regardless of the particular school of therapeutic technique used by the practitioner, the theoretical mechanism that the Krieger and Reiki systems work through, as well as the supposed benefits of these systems, remains essentially identical.

**TABLE 1**
**Distinctions between Reiki's and Therapeutic Touch**

| Characteristic | Reiki Healing Techniques | Krieger Therapeutic Touch |
|---|---|---|
| Description | A spiritual healing practice applicable for all religious traditions | A healing practice deduced from observing a spiritual healer applicable for all religious traditions |
| Touching | Passed through attunement | Learning of techniques |
| Mentoring | Reiki master lineage | Experienced practitioner |
| Intentionality | Allows the flow | Directs the flow |
| Techniques | Generally hands on; no assessment | Generally hands off; assessment |
| Outcomes | Relaxation, decreased anxiety, well-being, and healing | Relaxation, decreased anxiety, well-being, and healing |

### The Practice of Therapeutic Touch

TT is based on the belief that each living thing has a "life energy field" that extends beyond the surface of the body and generates an aura. Ideally, life energy flows freely to and from the environment. However, this balanced state is only maintained when people have unity with themselves. Practitioners of TT use their hands to direct the flow of chi so that the patient can heal faster. (Chi is the Chinese word used to describe "the natural energy of the universe". Chi is part of a metaphysical belief system. It permeates all things, including the human body.)

The aura of an individual can become unbalanced, misaligned, obstructed, or "out of tune". If desires are being repressed, rejected, fought, or frustrated, the individual falls out of harmony with the environment, either absorbing too much energy or releasing too much. Equally damaging, irregularities may develop in the individual's energy field that channel too much energy to one part of the body at the expense of others. The "unbalanced" energy field reflects its ailments in the physical body and impairs the natural, proper functioning of the human body. An unbalanced life energy field can result in the weakening of the immune system, a slowing down of the healing process, and/or various psychological disorders.

Therapists using TT believe that they can feel, manipulate, and "regulate" this life energy field by making movements that resemble massaging the air a few inches above the surface of the patient's body. These manipulations restore the energy field to a state of balance or harmony, to a proper alignment, or they may lead to an "unblocking" of a clog in the field or inevitably to transfer life energy from the therapist to the patient. This restoration of integrity to the field is thought to make it possible for the body to heal and operate at peak levels of efficiency. Put more directly, TT is a process by which energy is transmitted from one person (the practitioner) to another (the patient) for the purpose of enhancing the health process of one who is ill or injured[33].

For most schools of TT, the treatment generally involves four steps. These steps are: (1) engagement in a meditative process called "centering" that is thought to align the healer with the patient's energy level; (2) "assessment" of the forces emanating from the patient by using the hands of the practitioner; (3) "unruffling the field", which involves sweeping "stagnant energy" downward to prepare for the energy transfer, and (4) the transfer of the "energy" from practitioner to the patient.

## RESEARCH AND THERAPEUTIC TOUCH

Systematic research into the effects of TT has been going on for the past 35 years in some form. The research was dominated by quantitative methods up to the 1990s, but qualitative methods are now being used more often. Although proponents of TT refer to a voluminous and growing body of research**,** few studies have been well designed. In fact, the validity and reliability of the methods used in TT studies have been tested only sporadically regardless of the nature of the design. One of the biggest challenges to designing a valid study addressing the issue of effectiveness of TT is that touching is an inherently voluntary action. This means that it is difficult to design a study in such a way that participants are unaware of its purpose. Since the participants understand the purpose of the study, the outcomes are considered to be, at best, biased and, often, invalid.

The use of qualitative methods has increased alongside more traditional quantitative approach to studies of TT. However, the most popular method in touch studies has been that of nonparticipant observation[5,14,17,19,25,34,35,36]. For the past 2 decades, video recording has also been used increasingly in observational studies[15,17,18,26,27], illustrating efforts to develop a scientifically rigorous form of methodology through which the effectiveness of TT might be investigated. Despite these attempts, much of the research surrounding TT continues to be anecdotal.

There are inherent weaknesses involved in using anecdotal research to support the use of TT. Many studies investigating TT have involved small numbers of patients, thus precluding generalizations from the results. Anecdotal evidence supporting the use of TT includes a description of the process as well as results of the techniques, often presented from the patient's perspective, a method inherently rife with potential bias. Perhaps the most salient weakness is the inability to trace the sources of the patients' accounts. Patient confidentiality makes it impossible to contact specific patients regarding their view of the technique, thus making it difficult to validate the scientific quality of the account. Second, there is a lack of scientific control when using anecdotal evidence in research. Even if a certain subgroup of patients report increased levels of physical or mental healing from the use of TT, there is no scientific evidence to suggest that they would not have had the same experience without the intervention. One final weakness in relying on anecdotal research worth noting is the patient's expectation that healing will occur *because* TT has been used by the practitioner. This belief makes it impossible to rule out the placebo effect as the operative mechanism of the healing.

Although individual practitioners use TT in hospital settings, there are few formal evaluations found in the literature indicating its use in clinical settings. Some studies have opted to combine two methods, such as observation and interviews and observation and questionnaire[14,37], in order to compensate for the lack of a clinical setting. When available, however, such clinical studies have been effective exploring the efficacy of TT. One study conducted in a hospital setting involved an attempt to alter hemoglobin levels by using TT as the intervention. Because there are many elements (biological and environmental) that affect levels of hemoglobin, such studies have not been able to scientifically isolate TT as the precipitator of the change seen in hemoglobin levels of patients of any age[13]. There are, however, studies appearing in the literature whose designs make it possible to isolate the effects of TT.

A study by Newshan and Schuller-Civitella[37], also conducted in a clinical setting, demonstrated that TT is safe and effective when used with hospitalized patients. In fact, the authors found that the majority of in-patients (75%) who received TT as an intervention had a positive outcome It should be noted that the authors conducted a quality improvement study and did use a control group for comparison and confounded variables (such as medication). Data from this continuous quality improvement (CQI) clinical study suggest that TT, when provided in the clinical setting, promotes comfort, calmness, and well-being among hospitalized patients.

Clinical studies that investigate TT have often incorporated reliability and validity assessments[38]. Researchers who have combined study methods have documented the reliability of the Observation Schedule[39], the Touch Instrument[40], the Patient Touch Questionnaire[41], the Perception of Touch Instrument[42], and the Observation Instruments[17,26]. Reliability has been assessed using test-retests[41], panels of experts[17,42], Cohen's Kappa statistic[27,39], and Cronbach's alpha and Pearson's correlation coefficient[40]. Again, the majority of study outcomes indicate that the use of TT is thought to provide benefit to the majority of individuals who use it as a treatment modality.

The uses of TT as a healing modality in nonclinical settings are varied and numerous. However, practitioners are beginning to find that TT may be more effective in treating some disorders as compared to others. Among those conditions cited as particularly responsive to improvement using TT are pain[43,44,45], anxiety[43,49], and stress[36]. In addition, TT evokes the relaxation response[4], and it may promote wound healing[47,48].

TT significantly decreased pain and improved function in an elderly population with degenerative arthritis[49]. This study compared TT with progressive muscle relaxation in a sample of 128 subjects who served as their own controls. Data were collected over a 4-week period prior to the initiation of the experimental treatments and served as the baseline comparison against which results from the interventions were made. While reliable tools were used to measure pain and functional ability, five different TT practitioners provided the treatments, including the primary investigator. The TT practitioners' experiences were documented; however, participants were treated in the home environment, thus limiting control of extraneous variables. The study's limitations notwithstanding, participants who underwent TT had statistically significant decreases in pain and improvement in functional ability at the prescribed measurement times with little variation[49].

Finally, a study by Gordon et al.[45] utilized a single-blind, randomized control trial that used reliable measurement tools to measure the effects of pain in those with osteoarthritis. Three groups were created, including standard care, TT, and mimic touch. The same practitioners provided all treatments. The small sample size of 25 was a limitation of the study, and the findings cannot be generalized to all those with osteoarthritis. Despite these weaknesses, the author concluded that TT significantly decreased the pain in these elderly participants diagnosed with osteoarthritis.

## CONCLUSIONS

The growing popularity and use of TT is an issue that has generated controversy and concern within the medical community[50]. While anecdotal and traditional scientific evidence suggest that TT would be an advantageous addition for clinics and hospitals to include in their armamentarium of complementary interventions within the realm of traditional medicine, TT has not become widely available in the U.S. One reason for the lack of availability may be the dearth of conclusive scientific support for TT's efficacy and, therefore, its inclusion in clinic and hospital treatment planning would give it the appearance of legitimate practice which it may not yet deserve. Whether or not deserved, if TT were added to hospital and clinic treatment protocols without substantial scientific support, it would be thought to have the implicit support of the scientific community, at which point the question of its efficacy would be moot in the minds of many people; thus patients would utilize it, because they *believe* it works rather than *because* it works.

Another reason for the lack of availability of TT may be the inherent bias the traditional medical community tends to show with regard to holistic therapies. Nontraditional, holistic therapies are often looked on askance within the Western medical community as unproven, unhealthy, and fraudulent. Furthermore, the use of such therapies will often decrease the cost of medical services, therefore potentially decreasing the income of traditional medical providers.

Since TT has not yet been scientifically proven as per Western standards, leaders of the health care community are likely wary of lending support to TT at this time. If TT can be found to be a scientifically sound therapeutic technique, then it will be more readily accepted in the health care community. As the use of TT continues to increase by various health care professionals, clinics and hospitals have a difficult choice to make. These facilities may choose to include TT in the overall treatment planning provided that scientific monitoring governs its use and that this qualifying condition of use will alert patients that TT is not yet a scientifically accepted standard of practice. The pendulum, however, may swing the other way and clinics and hospitals may decide to not use TT under any circumstances, leaving a possibly extremely effective therapeutic tool at the fringes of traditional medicine instead of in a complementary role that it appears to deserve.

Even if TT cannot be quantitatively measured by any of the current methodologies supported by traditional scientists, it still may have a positive effect as the studies reviewed herein suggest. The mere acceptance of TT, even without traditional scientific evidence attesting to its effectiveness, means that patients are asserting themselves and taking some level of responsibility for their own health outcomes. From their perspective, TT is economically advantageous for clinics and hospitals to include in their treatment planning protocols. Otherwise, patients requesting TT as part of their treatment will go elsewhere to get their needs met. If those health care practitioners that use TT have no greater power than to produce healthy, positive outlooks among patients that are ill, then they serve an important role in the current medicalized system of care in the U.S. and elsewhere.
